# Supplementary material for: High-throughput screening of prostate cancer risk loci by single nucleotide polymorphisms sequencing
Source: Nat Commun. 2018 May 22;9:2022. doi: 10.1038/s41467-018-04451-x (PMC5964124; doi:10.1038/s41467-018-04451-x)
Supplement: Supplementary file 1 — Supplementary Information [file 41467_2018_4451_MOESM1_ESM.pdf]

Supplementary Information

High-throughput screening of prostate cancer risk loci by single nucleotide polymorphisms sequencing

Peng Zhang, Ji-Han Xia, Jing Zhu, Ping Gao, Yi-Jun Tian, Meijun Du, Yong-Chen Guo, Sufyan Suleman, Qin Zhang, Manish Kohli, Lori S. Tillmans, Stephen N. Thibodeau, Amy J. French, James R. Cerhan, Li-Dong Wang, Gong-Hong Wei and Liang Wang

Supplementary Figures 1-10

Supplementary Tables 1-5

Supplementary References

Supplementary Figures

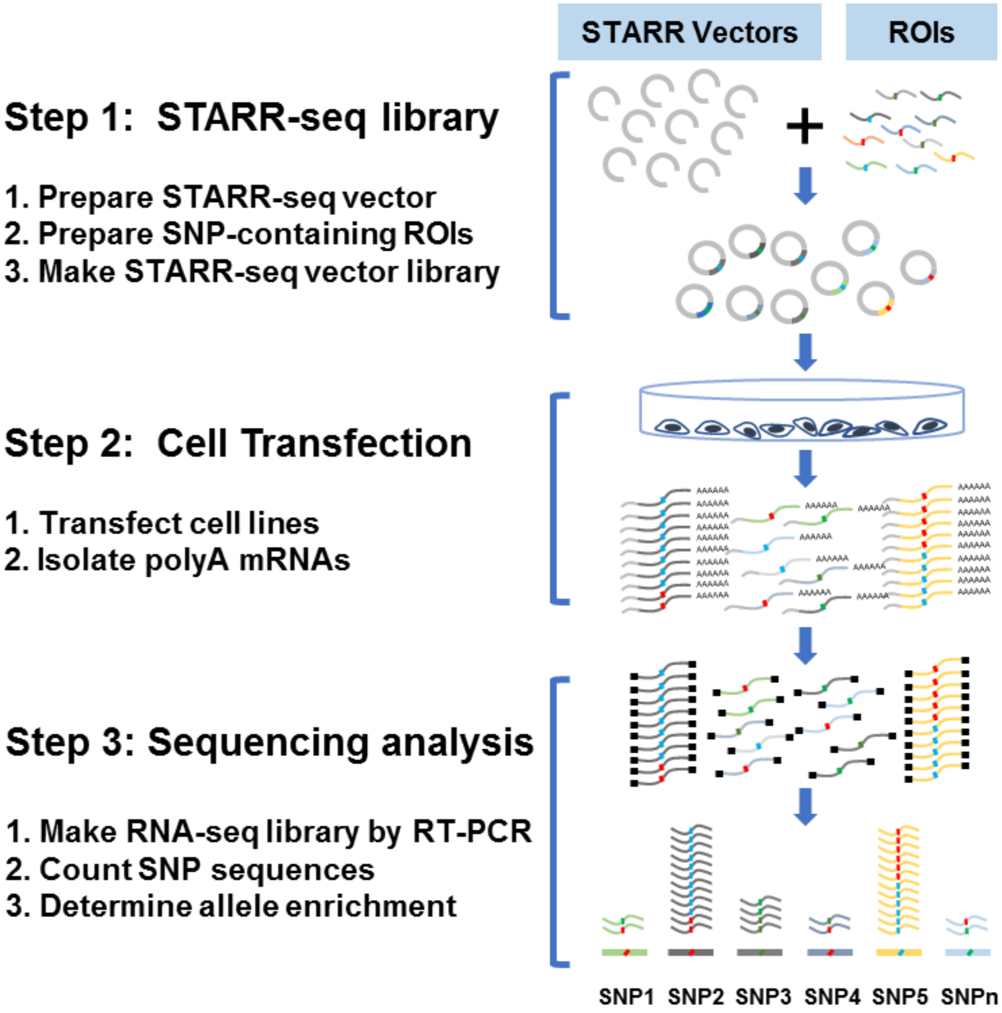

Supplementary Figure 1
Workflow of STARR-seq.
Three key steps for STARR-seq include STARR-seq library preparation, cell transfection, and RNA-seq library preparation and analysis. ROI: regions of interest.

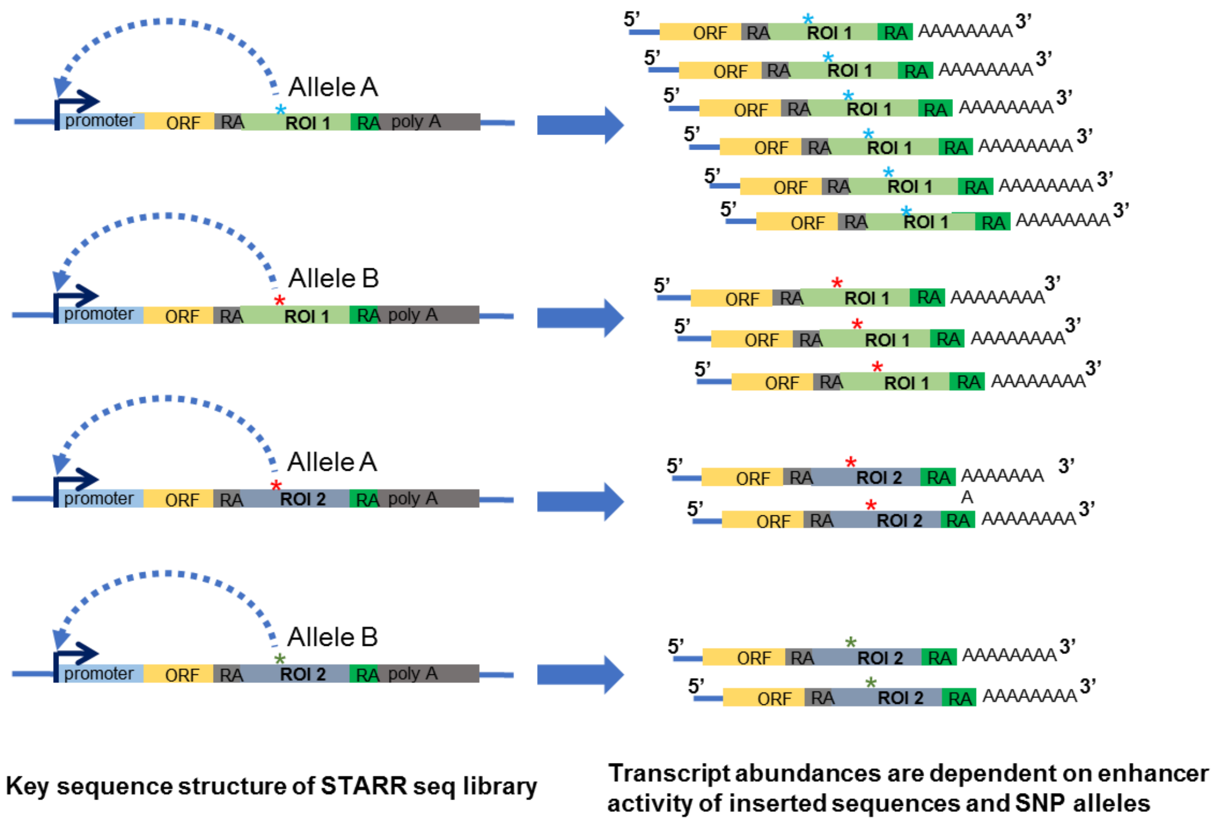

Supplementary Figure 2

Key elements of STARR-seq vector and allele-dependent transcription.

The key elements include super core promoter 1, ORF for green fluorescent protein, recombination arm (AR), region of interest (ROI), recombination arm (AR) and poly A tail. Both alleles (A and B) in two regions of interest (ROI 1 and ROI 2) are shown. For ROI 1, allele A shows stronger enhancer activity than allele B. For ROI 2, both alleles A and B have similar enhancer activity. Star (*) indicates SNP location.

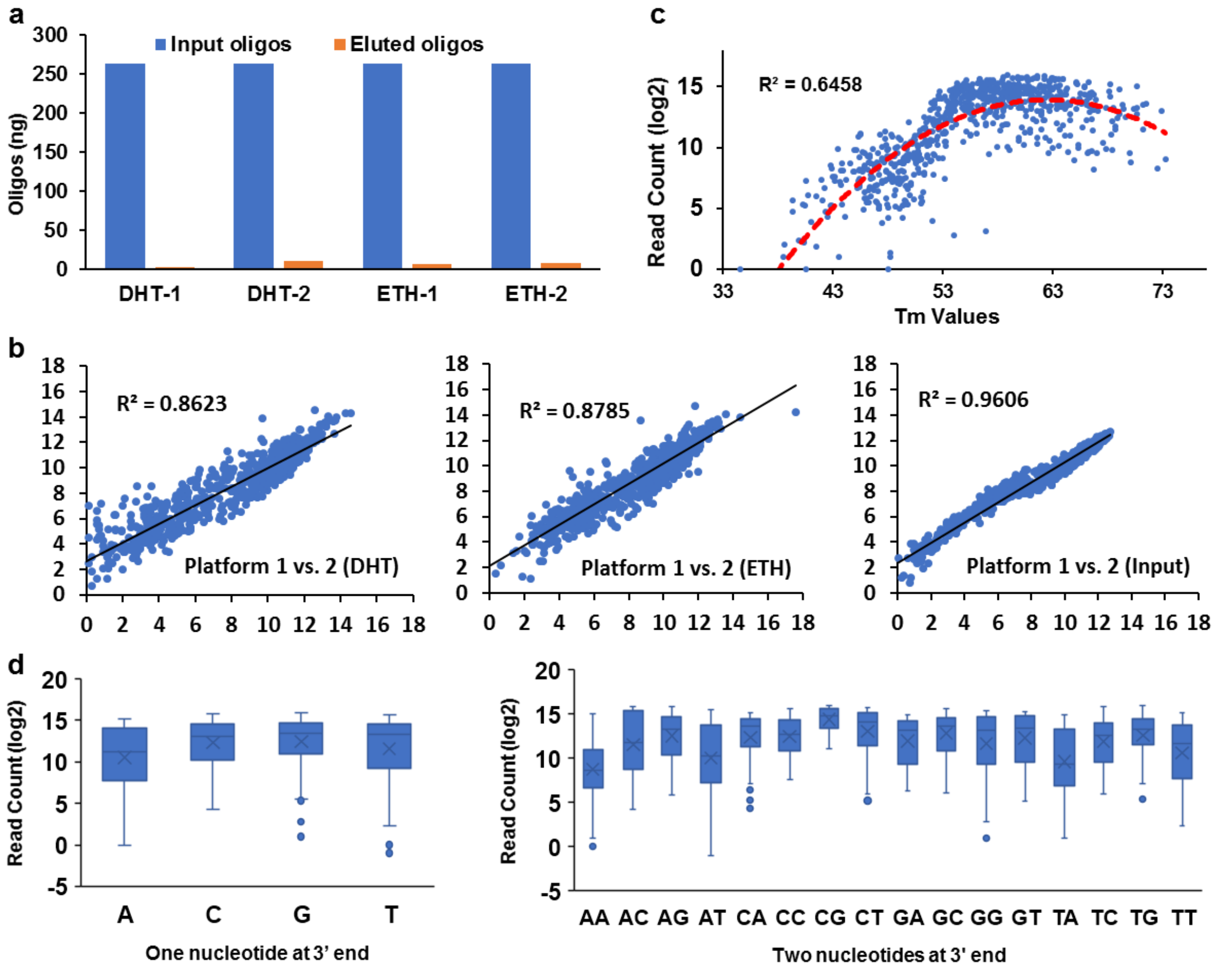

Supplementary Figure 3

QC analysis and effect of oligo content on sequencing read counts.

(a) Protein-bound oligos accounted for 2-4% of inputs and were recovered in elution buffer. **(b)** Correlation between different platforms (Affymetrix and Signosis) in SNPs-seq. Mapped read counts were first transformed to log2 values, and then plotted along x (platform 1-Affymetrix) and y (platform 2-Signosis) axis. **(c)** Association of Tm values with read counts. Lower Tm values contribute to lower read counts. **(d)** Association of nucleotides at 3' end with read counts. The nucleotides A, T, AA, AT, TA or TT at 3' end of tested oligos are attributable to lower read counts. The upper, middle, and lower bounds of boxes represent the 75th, 50th, and 25th percentile of the values, respectively. The whiskers represent 95th to 5th percentile.

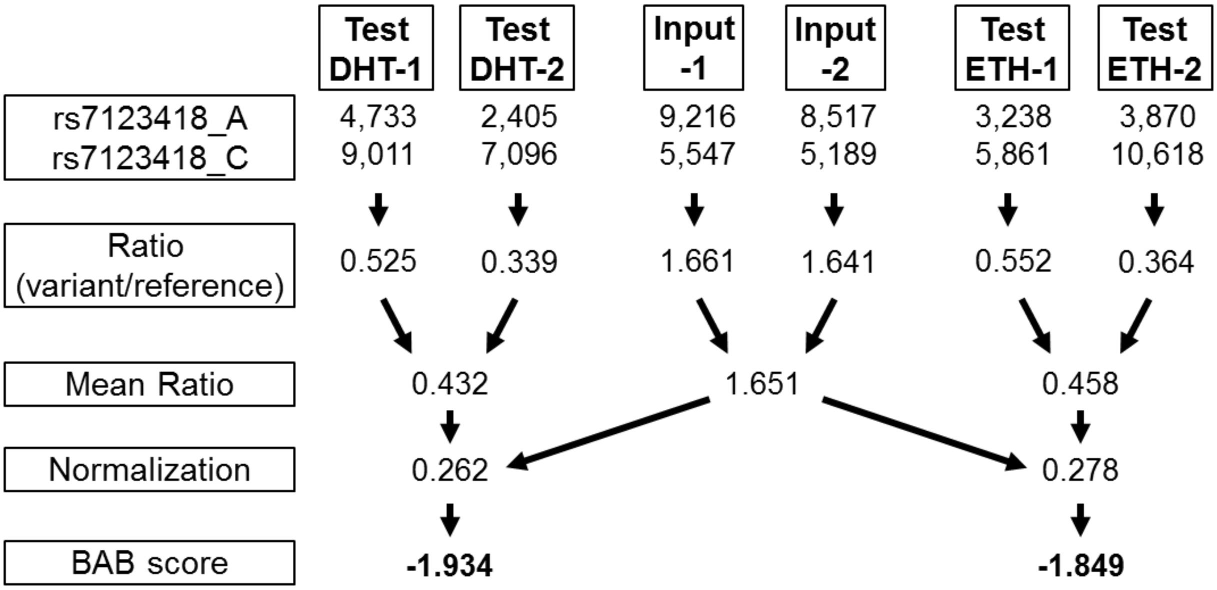

Supplementary Figure 4

Example of the BAB score calculation at SNP rs7123418 site.

Step 1: generate ratio by dividing variant allele count to reference allele count. Step 2: calculate mean ratio of the technical replicates for each group. Step 3: normalize the mean ratio of test group to input control group. Step 4: generate BAB score by log2 transformation of the normalized ratio.

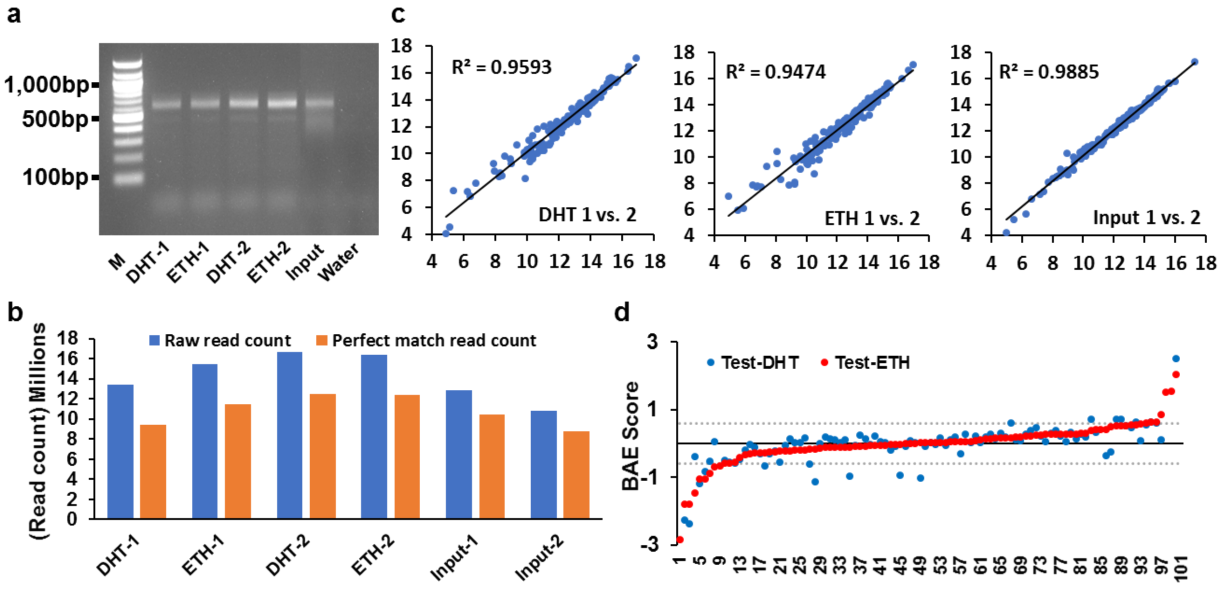

Supplementary Figure 5

STARR-seq library QC analysis and BAE score distribution.

(a) Both test and input control samples show a range of 500-700 bp length in 1.5% agarose gel. **(b)** Mapping of read count from STARR-seq. The percentage of mapped read count is ~70% in test samples and ~81% in input controls. **(c)** Correlations between technical replicates. Mapped read counts were first transformed to log2 values and then plotted along x (replicate 1) and y (replicate 2) axis. **(d)** Overall distribution of BAE scores among the 101 selected SNPs. The red line represents BAE scores in ETH group while the blue dots represent the corresponding BAE scores from the DHT group.

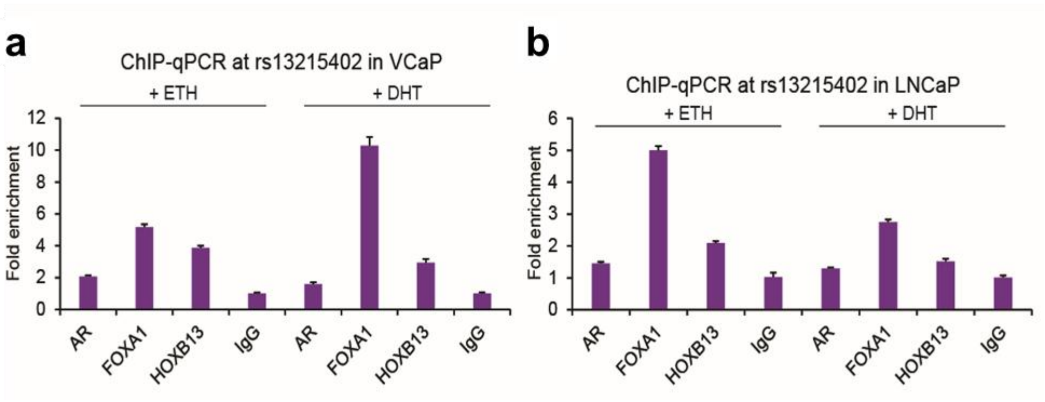

Supplementary Figure 6

ChIP-qPCR analysis at rs13215402 site.

ChIP-qPCR analyses show the binding of AR, FOXA1 and HOXB13 at rs13215402 region in VCaP **(a)** and LNCaP cells **(b)** respectively. Mean ± s.d.. Compared to IgG, chromatin binding of AR, FOXA1, and HOXB13 is enriched at rs13215402-containing region.

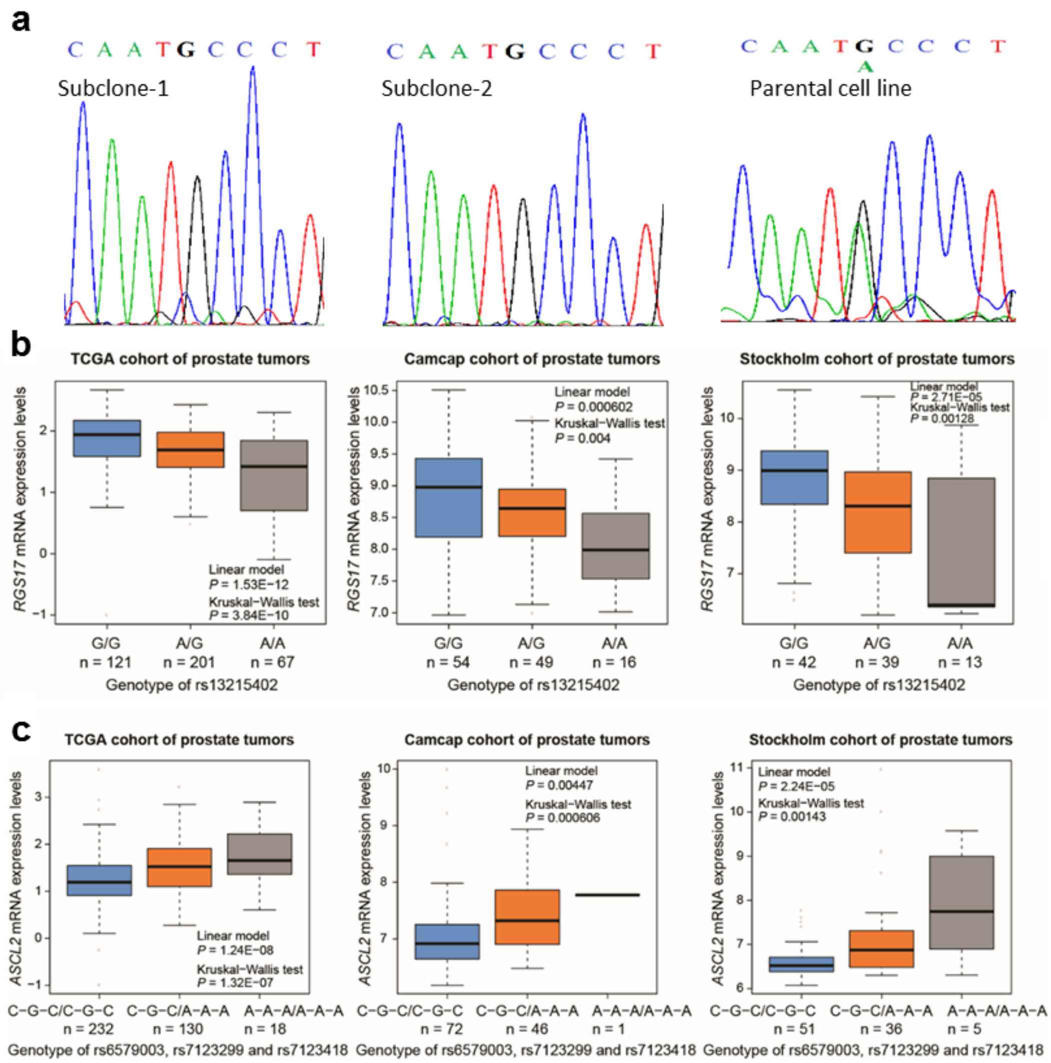

Supplementary Figure 7

Additional SNP genotypes and target gene expression.

(a) Sequencing analysis confirmed conversion of heterozygous G/A to homozygous G/G at rs13215402 in 22Rv1 cell line. **(b)** Association of rs13215402 genotypes and *RGS17* expression. Compared to G/G genotype, the rs13215402 A/A genotype is associated with lower *RGS17* expression in TCGA, Camcap and Stockholm cohorts of prostate cancers[1], respectively. The upper, middle, and lower bounds of boxes represent the 75th, 50th, and 25th percentile of the values, respectively. The whiskers represent 95th to 5th percentile. The *P* values were examined by liner regression and the Kruskal–Wallis H test, respectively. **(c)** Association of haplotype genotypes (rs6579003, rs7123299, and rs7123418) and *ASCL2* expression. The haplotype genotype AAA/AAA confers higher *ASCL2* expression than haplotype CGC/CGC in TCGA, Camcap and Stockholm cohorts of prostate cancers, respectively. The *RGS17* and *ASCL2* mRNA levels were assessed by RNA-seq in a collection of 389 (TCGA), and Illumina Expression BeadChip-based transcriptional profiling of 119 (Camcap) and 94 (Stockholm) human prostate tissue samples, respectively. The upper, middle, and lower bounds of boxes represent the 75th, 50th, and 25th percentile of the values, respectively. The whiskers represent 95th to 5th percentile. The *P* values were examined by liner regression and the Kruskal–Wallis H test, respectively.

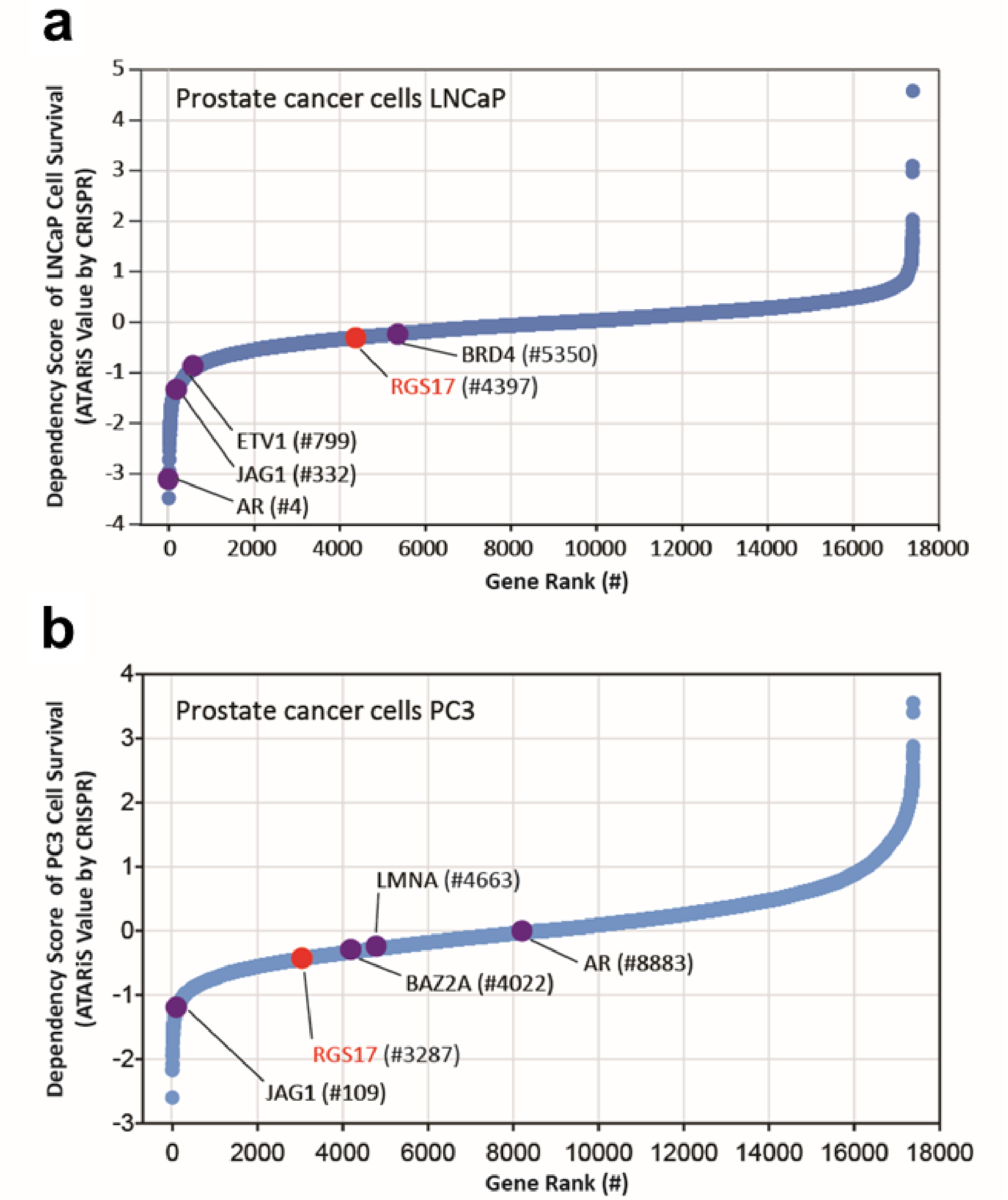

Supplementary Figure 8

Association of *RGS17* with prostate cancer cell survival.

Genome-wide loss-of-function screening of the genes that are essential for the survival of the AR-positive prostate cancer cell line LNCaP **(a)**, and the AR-negative prostate cancer cell line PC3 **(b)**. Note that lower ATARiS values showed an elevated dependency of the cells on the indicated genes. The genes *AR*, *JAG1*, *ETV1*, and *BRD4* are known to be important for LNCaP cancer cell growth and survival[2, 3, 4, 5], and *JAG1*, *BAZ2A*, and *LMNA* are known for PC3 cell proliferation and survival[5, 6, 7]. As expected, AR was not essential for the survival of PC3. Note that the essentiality of *RGS17* is strikingly higher than *BRD4* in LNCaP **(a)**, and *BAZ2A* and *LMNA* in PC3 cells **(b).**

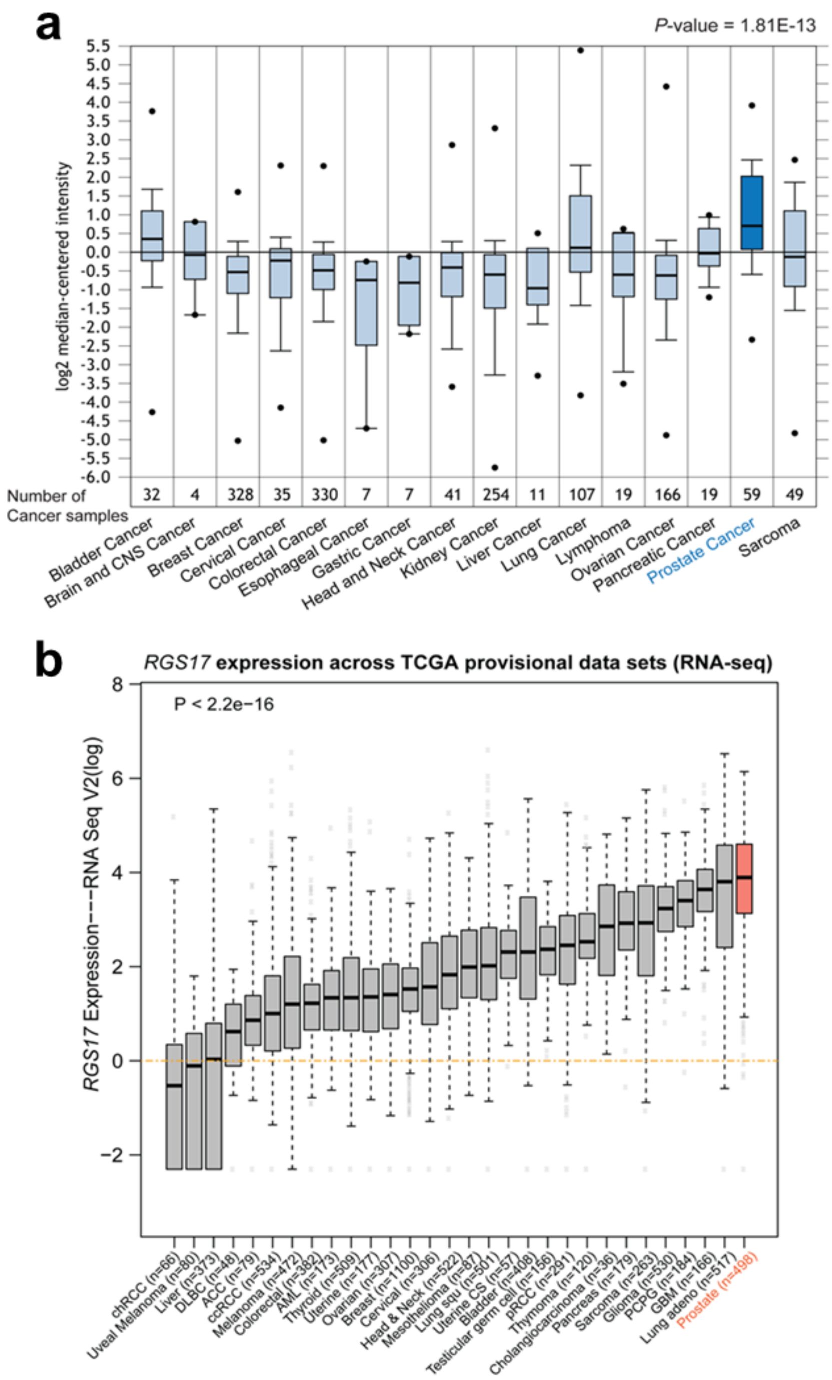

Supplementary Figure 9

Expression of *RGS17* in different cancer types.

RGS17 expression level is significantly higher in prostate cancer than other type of cancers in 1,468 samples[8] with cDNA microarray-based expression profiling **(a)**, and 9,121 samples[9] transcriptionally profiled by RNA-seq **(b)**. The upper, middle, and lower bounds of boxes represent the 75th, 50th, and 25th percentile of the values, respectively. The whiskers represent 95th to 5th percentile. The *P* values were calculated using the non-parametric Kruskal-Wallis H test.

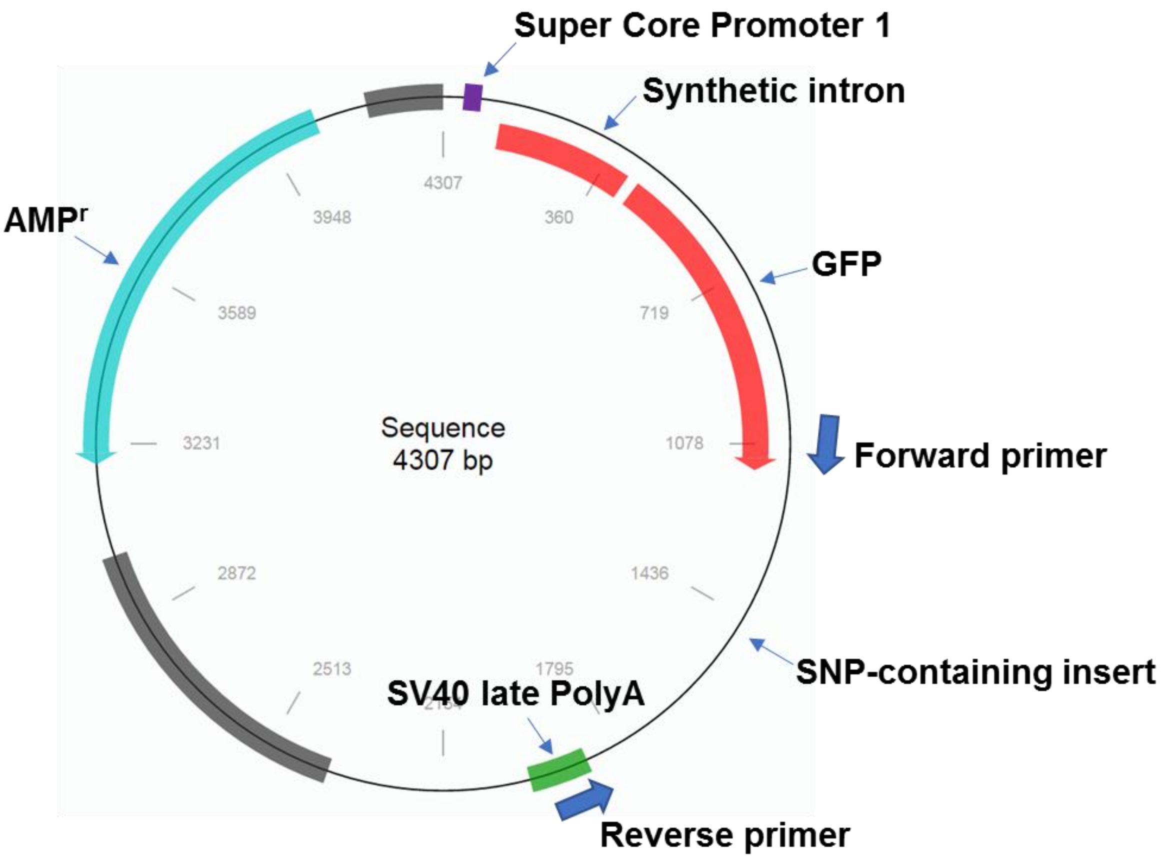

Supplementary Figure 10
Mapping of STARR-seq reporter-specific primers.
Two reporter-specific primers were used to amplify the SNP-containing sequences for STARR-seq library preparation.

Supplementary Tables

Supplementary Table 1. The buffers used for ChIP.

Buffers	Composition
Hypotonic lysis buffer	20 mM Tris-Cl at pH 8.0, 10% glycerol, 10 mM KCl, 2 mM DTT, and complete Protease Inhibitor cocktail
SDS lysis buffer	50 mM Tris-HCl at pH 8.1, 10 mM EDTA, 0.5% SDS, and complete Protease Inhibitor
Blocking buffer	0.5% BSA in IP buffer
IP buffer	20 mM Tris-HCl, pH8.0, with 2 mM EDTA, 150 mM NaCl, 1% Triton X-100, and Protease Inhibitor cocktail
Wash buffer I	20 mM Tris-HCl at pH 8.0, 2 mM EDTA, 0.1%SDS, 1% Triton X-100, and 150 mM NaCl
Wash buffer II	20 mM Tris-HCl at pH 8.0, 2 mM EDTA, 0.1% SDS, 1% Triton X-100, and 500 mM NaCl
Wash buffer III	10 mM Tris-HCl at pH 8.0, 1 mM EDTA, 250 mM LiCl, 1% Deoxycholate, and 1% NP-40
Wash buffer IV	10 mM Tris-HCl at pH 8.0, and 1 mM EDTA
Extraction buffer	10 mM Tris-HCl at pH 8.0, 1 mM EDTA, and 1% SDS

Supplementary Table 2. The antibodies used for ChIP.

Antibodies	Cat. No.	Manufacturer
AR	sc-816x	Santa Cruz Biotechnology
HOXB13	sc-66923x	Santa Cruz Biotechnology
FOXA1	ab23738	Abcam
IgG	sc-2027x	Santa Cruz Biotechnology

Supplementary Table 3. The primers used for ChIP-qPCR and allele-specific qPCR.

Assays	Primer name	Sequence (5´-3´)
ChIP-qPCR		
at rs13215402	rs13215402ChIP-79F	AGGTTGGAAAACTCAGGGGTG
	rs13215402ChIP-79R	GTGGCTAAACCAGAAAAGCTGGA
ChIP-AS-qPCR		
at 13215402	rs13215402A-F	AGCAGTATATGATGAACCCCAATA
	rs13215402G-F	AGCAGTATATGATGAACCCCAATG
	rs13215402-96R	GCAAACAAGGAAAGGAAATGCTCA
ChIP-AS-qPCR		
at rs7123299	rs7123299A-F	CCGGCCTCTGTGCCTGGGCA
	rs7123299G-F	CCGGCCTCTGTGCCTGGGCG
	rs7123299-217R	AGGGGCCTGTGCAGGGTCTG

Supplementary Table 4. sgRNA oligos and *RGS17* RT-qPCR primers

**Sequence names**	**Sequences (5'-3')**
sgRNA oligos	
rs13215402G-top	CACCGTATGATGAACCCCAATGCCC
rs13215402G-bottom	AAACGGGCATTGGGGTTCATCATAC
rs13215402A-top	CACCGTATGATGAACCCCAATACCC
rs13215402A-bottom	AAACGGGTATTGGGGTTCATCATAC
Repair template	TGTAAAGGTTGGAAAACTCAGGGGTGTAGCAGTATATGAT GAACCCCAATA/GCCCTGGCTGATCCAGCTTTTCTGGTTTAG CCACAGTGATCTTTGTCACTG
RT-qPCR primers	
RGS17-F	GATGATGAAGGCCCCAGCAG
RGS17-R	TGTGAGGATTGGGATCCAACAG
ACTB-F	AGAAAATCTGGCACCACACC
ACTB-R	AGAGGCGTACAGGGATAGCA

Supplementary Table 5. siRNAs used for gene knockdown assay.

**Product name**	**Cat. No.**	**Target sequence**	**Lot No.**
Hs_RGS17_siRNA1	SI02639644	CAGGAGAGTTTACTATGCTAA	201612210015
Hs_RGS17_siRNA2	SI03050803	ATGGAGAGTATCCAGGTCCTA	201612210014

Supplementary References

1. Whitington T*, et al.* Gene regulatory mechanisms underpinning prostate cancer susceptibility. *Nat Genet* **48**, 387-397 (2016).

2. Aguirre AJ*, et al.* Genomic Copy Number Dictates a Gene-Independent Cell Response to CRISPR/Cas9 Targeting. *Cancer Discov* **6**, 914-929 (2016).

3. Asangani IA*, et al.* Therapeutic targeting of BET bromodomain proteins in castration-resistant prostate cancer. *Nature* **510**, 278-282 (2014).

4. Tomlins SA*, et al.* Distinct classes of chromosomal rearrangements create oncogenic ETS gene fusions in prostate cancer. *Nature* **448**, 595-599 (2007).

5. Yu Y*, et al.* Androgen receptor promotes the oncogenic function of overexpressed Jagged1 in prostate cancer by enhancing cyclin B1 expression via Akt phosphorylation. *Mol Cancer Res* **12**, 830-842 (2014).

6. Gu L*, et al.* BAZ2A (TIP5) is involved in epigenetic alterations in prostate cancer and its overexpression predicts disease recurrence. *Nat Genet* **47**, 22-30 (2015).

7. Kong L, Schafer G, Bu H, Zhang Y, Zhang Y, Klocker H. Lamin A/C protein is overexpressed in tissue-invading prostate cancer and promotes prostate cancer cell growth, migration and invasion through the PI3K/AKT/PTEN pathway. *Carcinogenesis* **33**, 751-759 (2012).

8. Rhodes DR*, et al.* ONCOMINE: a cancer microarray database and integrated data-mining platform. *Neoplasia* **6**, 1-6 (2004).

9. Cerami E*, et al.* The cBio cancer genomics portal: an open platform for exploring multidimensional cancer genomics data. *Cancer Discov* **2**, 401-404 (2012).
