# Supplementary material for: High-throughput screening of prostate cancer risk loci by single nucleotide polymorphisms sequencing
Source: Nat Commun. 2018 May 22;9:2022. doi: 10.1038/s41467-018-04451-x (PMC5964124; doi:10.1038/s41467-018-04451-x)
Supplement: Supplementary file 3 — Description of Additional Supplementary Files [file 41467_2018_4451_MOESM3_ESM.pdf]

Description of Additional Supplementary Files

File Name: Supplementary Data 1
Description: 374 SNPs and their chromosome positions, lead SNPs, eQTL P values and ChIP-seq scores.

File Name: Supplementary Data 2
Description: Read counts in 20 separate SNPs-seq assays.

File Name: Supplementary Data 3
Description: BAB score of 101 selected SNPs and primer sequences for STARR-seq.

File Name: Supplementary Data 4
Description: STARR-seq read counts and BAE score for 101 SNPs.

File Name: Supplementary Data 5
Description: Time and cost estimation for 20 SNPs-seq tests.
